# Chitosan Versus Dapagliflozin in a Diabetic Cardiomyopathy Mouse Model

**DOI:** 10.3390/ijms25042118

**Published:** 2024-02-09

**Authors:** Georgică Târtea, Aurel Popa-Wagner, Veronica Sfredel, Smaranda Ioana Mitran, Alexandra Oltea Dan, Anca-Maria Țucă, Alexandra Nicoleta Preda, Victor Raicea, Eugen Țieranu, Dragoș Cozma, Radu Vătășescu

**Affiliations:** 1 Department of Physiology, University of Medicine and Pharmacy of Craiova, 200349 Craiova, Romania; georgetartea@gmail.com (G.T.); veronicasfredel@yahoo.com (V.S.); smamican@yahoo.com (S.I.M.); puiu.alexandra.oltea@gmail.com (A.O.D.); ancabirau94@yahoo.com (A.-M.Ț.); 2 Department of Neurology, University Hospital Essen, University of Duisburg-Essen, 45147 Essen, Germany; 3 Department of Cardiology, University of Medicine and Pharmacy of Craiova, 200349 Craiova, Romania; dr.raicea.victor@gmail.com (V.R.); tieranueugen@gmail.com (E.Ț.); 4 Department of Cardiology, “Victor Babes” University of Medicine and Pharmacy, 2 Eftimie Murgu Sq., 300041 Timisoara, Romania; dragoscozma@gmail.com; 5 Cardio-Thoracic Pathology Department, “Carol Davila” University of Medicine and Pharmacy, 050474 Bucharest, Romania; radu_vatasescu@yahoo.com

**Keywords:** chitosan, dapagliflozin, diabetes mellitus, cardiomyopathy

## Abstract

Diabetes mellitus is a metabolic disorder with global economic implications that can lead to complications such as diabetic cardiomyopathy. The aim of this study was to compare the effects of chitosan versus dapagliflozin in mouse diabetic cardiomyopathy. We used 32 C57Bl/6 male mice aged between 8 and 10 weeks, which were randomly divided into Control—without diabetes mellitus (DM), type 1 DM (T1DM), T1DM + Chitosan, and T1DM + Dapapgliflozin groups. We induced diabetes with streptozotocin and treated the animals for 12 weeks. The analysis showed a reduction in intramyocardial fibrosis in the T1DM + Dapapgliflozin compared to T1DM animals. In T1DM + CHIT, a reduction in intramyocardial fibrosis was observed although, accordingly, there was also no significant decrease in blood glucose. The level of oxidative stress was reduced in the groups of treated animals compared to T1DM. All these observed changes in the structure and function of hearts were highlighted in the echocardiographic examination. In the treated groups, there was delayed appearance of left ventricular (LV) hypertrophy, a slight decrease in the ejection fraction of the LV, and an improved diastolic profile. The results demonstrate that chitosan has promising effects on diabetic cardiomyopathy that are comparable to the beneficial effects of dapagliflozin.

## 1. Introduction

Diabetes mellitus is one of the most well-known metabolic disorders worldwide and is characterized by persistently high blood glucose levels (hyperglycemia) with a major impact on health [1]. According to the classical definition, diabetes mellitus occurs mainly from either a lack of insulin secretion (type 1 diabetes mellitus (T1DM)) or a malfunction in insulin action, especially through the development of insulin resistance that affects the liver and peripheral tissues (type 2 diabetes (T2DM)) [1]. According to the latest IDF (International Diabetes Federation) report, the estimated global incidence of people with diabetes is 537 million [2], represented by approximately 1 in 10 adults between the ages of 20 and 79 [2]. Moreover, an additional 240 million adults (aged 20–79 years) globally have impaired glucose tolerance (two-hour glucose levels of 140 to 199 mg per dL [7.8 to 11.0 mmol] based on the 75 g oral glucose tolerance test), a condition that indicates a higher risk of developing diabetes [2]. Crucially, because of the long-term complications that result from uncontrolled high blood sugar levels, diabetes places substantial financial strain on a global scale [1]. According to the IDF, approximately 10% of the global health expenditure is allocated to diabetes. The total annual economic cost of diabetes is estimated to exceed USD 760 billion globally, with the United States alone representing an annual cost of USD 412.9 billion [3]. One of the most frequent complications of diabetes is diabetic cardiomyopathy [4,5]. In this study, our focus was on T1DM induced by streptozotocin. This model mimics the absence of insulin and subsequent hyperglycemia observed in natural T1DM. However, it achieves this effect through a distinct mechanism, not via autoimmune-mediated destruction of beta cells, as seen in human T1DM.

Diabetic cardiomyopathy is defined as the pathophysiological evolution of the heart in the context of diabetes and is characterized by parietal hypertrophy and diastolic dysfunction in the early stages progressing to a reduction in the ejection fraction in the late stages, all in the absence of coronary disease or other secondary causes [4]. Although myocardial damage is produced by persistently elevated blood glucose levels and all its subsequent consequences, there are other mechanisms that may contribute to the decreased performance of the diabetic heart, such as altered Ca^2+^ handling in the cytosol or in the mitochondria of cardiomyocytes [6]. The unclarity in the data also extends to knowledge regarding the therapeutic target effect of various molecules, such as chitosan and dapagliflozin.

Chitosan, which is abundantly found in the shells of crustaceans and insects, as well as in the cell walls of fungi, is obtained by deacetylating chitin and is the second most abundant polymer in nature after cellulose [7,8,9]. Chitosan cannot be degraded in the gastrointestinal tract due to the absence of digestive enzymes from the digestive tract of humans and animals involved in its the digestion [7]. Moreover, chitosan is absorbed at the level of the intestinal mucosa, and in vivo studies have shown that the absorption of chitosan is inversely proportional to its molecular weight [10,11]. Previous studies have demonstrated that chitosan reduces plasma cholesterol levels by promoting fecal fat excretion [12]. There are multiple studies documenting that chitosan exhibits antioxidative, anti-inflammatory, antimicrobial, hypolipidemic, antiobesity, hypoglycemic, and antihypertensive properties [7]. Chitosan improves the pathophysiology of diabetes via multiple pathways. One of these mechanisms is to improve muscle absorption of glucose by facilitating the movement of glucose transporter 4 (GLUT4) from the cytoplasm to the cell membranes using chitosan, as demonstrated in rats with T1DM induced by STZ [13]. Chitosan also exerts antidiabetic effects by inhibiting intestinal enzymes that hydrolyze carbohydrates, reducing carbohydrate digestion and absorption, inhibiting hepatic gluconeogenesis by upregulating hepatic leptin receptor-b (LepRb) expression and activating downstream JAK2-STAT3 signaling, protecting pancreatic β-cells, and preventing STZ-induced apoptosis, or it may facilitate increased glucose uptake by regulating adiponectin expression through the peroxisome proliferator-activated receptor (PPAR)-γ in adipocytes [13,14]. Last but not least, chitosan exerts prebiotic effects, positively modulating the gut microbiota [15]. This has attracted attention more recently. Chitosan, similar to galacto-oligosaccharide, inulin, and beta-glucan, increases the Firmicutes/Bacteroidetes ratio. This leads to the upregulation of Verrucomicrobiales or the downregulation of Burkholderiales [15]. Through this manipulation of the gut microbiota, chitosan exerts effects that reduce the progression of diabetes [15]. On the other hand, dapagliflozin is one of the newest molecules used in the treatment of diabetes, and it can prevent or alleviate induced complications [16]. Dapagliflozin belongs to the class of sodium glucose cotransporter 2 inhibitors (SGLT2I). Sodium glucose cotransporter 2 inhibitors (SGLT2I) work by directly targeting the proximal tubule in the kidney [16], which is achieved by blocking the sodium glucose transporter, which in turn reduces glucose reabsorption by the kidneys [16]. As a result, these inhibitors enhance the excretion of glucose through urine [17]. These medications have been prioritized for the treatment of diabetes in patients with cardiovascular disease [16,18]. Although dapagliflozin has a completely different mechanism of action from chitosan, we chose to analyze the target effects of these two drugs on the hearts of mice with streptozotocin-induced diabetes. Furthermore, we opted to analyze dapagliflozin because SGLT2 inhibitors (SGLT2Is) have demonstrated their effectiveness in safeguarding against cardiomyopathy compared to other traditional antidiabetic medications. This has granted SGLT2Is the status of a guideline recommendation for patients with heart failure with preserved ejection fraction. The diabetic cardiomyopathy utilized in our study serves as a model for this human pathology.

In this study, we aimed at the comparative analysis of chitosan—a food additive approved both in the United States of America and in Europe for its multiple effects—with dapagliflozin—a new antidiabetic compound whose molecular mechanisms in diabetes are well known—to evaluate their effectiveness as preventive drugs in cardiomyopathy produced by diabetes mellitus.

## 2. Results

### 2.1. Analysis of Blood Glucose and Weight of the Animals

**Figure 1:**
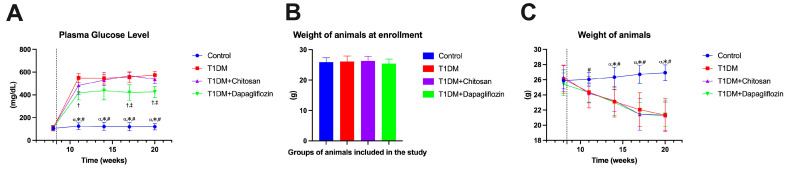
Assessment of the blood glucose levels as well as weight of animals before and after the administration of streptozotocin (STZ). Data are shown as mean ± S.D. (**A**) Plasma glucose concentration. (**B**) Weight of the animals at time of enrollment in the study. (**C**) Evolution of animal weight after onset of diabetes mellitus. The vertical dotted line represents the administration of STZ. α *p* < 0.05 vs. T1DM, * *p* < 0.05 vs. T1DM + Chitosan, # *p* < 0.05 vs. T1DM + Dapagliflozin, † *p* < 0.05 vs. T1DM, and ‡ < 0.05 vs. T1DM + Chitosan.

Unlike sodium chloride treatment, streptozotocin rapidly induced hyperglycemia in mice beginning at 3–5 days after intraperitoneal injection (Figure 1A). Applying dapagliflozin treatment reduced blood glucose levels compared to the T1DM and T1DM + Chitosan groups. Details of the plasma glucose values and the ANOVA test results can be found in the additional materials in Tables S1 and S2.

### 2.2. Body Weight Assessment of Animals

The recording of the initial weight of the animals showed there were comparative values between the groups (Figure 1B, Table S3). After the onset of diabetes, a significant decrease in weight was observed in all STZ-injected animals, regardless of the administered therapeutic protocol (chitosan or dapagliflozin), as shown in Figure 1C.

### 2.3. Effect on Lipid Properties

**Figure 2:**
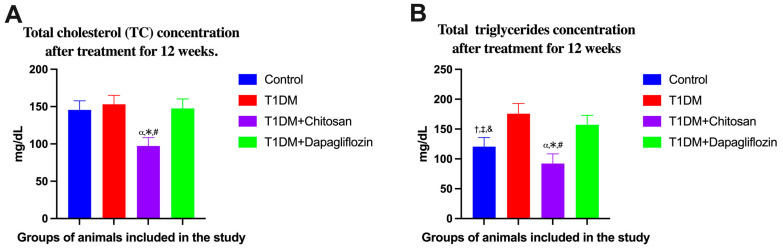
Effect of chitosan and dapagliflozin on total cholesterol (**A**) and triglycerides (**B**) after treatment for 12 weeks. Data are shown as mean ± S.D. α *p* < 0.05 vs. Control, * *p* < 0.05 vs. T1DM, # *p* < 0.05 vs. T1DM + Dapagliflozin, † *p* < 0.05 vs. T1DM, ‡ *p* < 0.05 vs. T1DM + Chitosan, and & *p* < 0.05 vs. T1DM + Dapagliflozin.

It should be mentioned that both total cholesterol and triglycerides were analyzed only once, at the end of the experimental study. A slight increase in cholesterol and triglyceride levels was observed in the group of animals with diabetes mellitus (T1DM) compared to the Control group. This increase was also observed in the T1DM + Dapagliflozin group, but a significant decrease in these parameters was observed in the T1DM + Chitosan group. Moreover, in all groups of animals, no pathological increase in cholesterol was observed (Figure 2A). Regarding triglycerides, a concentration of 120.5 ± 15.21 mg/dL was recorded in the Control group (without diabetes) triglycerides compared to the higher concentration of 175.8 ± 17.15 mg/dL recorded for the T1DM group without treatment. An increased concentration of triglycerides, 156.9 ± 16.08 mg/dL, was also recorded on the T1DM + Dapagliflozin group, whereas a much lower concentration (92.25 ± 16.18 mg/dL) was recorded in the T1DM + Chitosan group than in the other groups (Figure 2B). The results of the ANOVA test for cholesterol are shown in Table S4 and for triglycerides in Table S5.

### 2.4. Effect of Chitosan and Dapagliflozin on LV Dimensions and Functions

**Figure 3:**
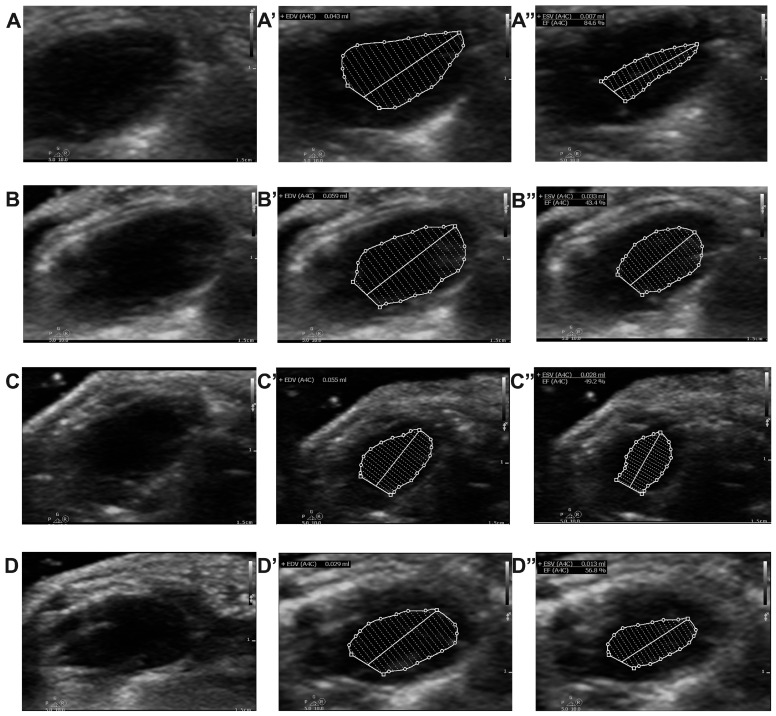
Representative images used for calculating the left ventricular ejection fraction (LVEJ) at the end of the study in mice without diabetes in the Control group (**A**), untreated mice in the T1DM group with diabetes (**B**), mice in the diabetic group treated with chitosan (T1MD + Chistosan) (**C**), and mice in the diabetic group treated with dapagliflozin (T1DM + Dapagliflozin) (**D**). The capital letters with (’) indicate the calculation of the end-diastolic volume of the left ventricle. The capital letters with (”) indicate the calculation of the end-systolic volume of the left ventricle as well as the automatic determination of LVEF.

**Figure 4:**
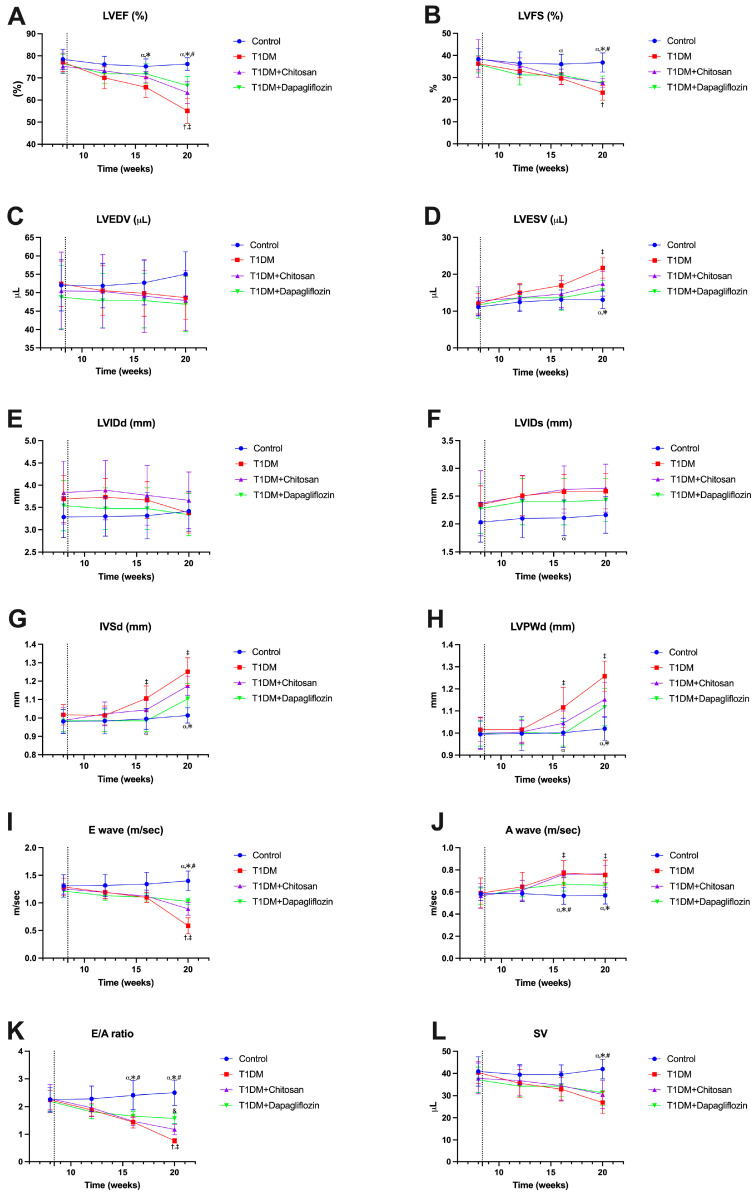
Echocardiographic parameters evaluated in our study before administration of streptozotocin (week 8) and after administration of streptozotocin (weeks 12, 16, and 20). (**A**) Left ventricular ejection fraction (LVEF). (**B**) Fractional shortening (FS). (**C**) Left ventricle (LV) end-diastolic volume (LVEDV). (**D**) LV end-systolic volume (LVESV). (**E**) LV internal diastolic diameter (LVIDd). (**F**) LV internal systolic diameter (LVISd). (**G**) Interventricular septal width during end-diastole (IVSd). (**H**) LV posterior wall width during end-diastole (LVPWd). (**I**) E wave (early diastole). (**J**) A wave (atrial systole). (**K**) E/A ratio. (**L**) stroke volume (SV). α *p* < 0.05 vs. T1DM, * *p* < 0.05 vs. T1DM + Chitosan, # *p* < 0.05 vs. T1DM + Dapagliflozin, † *p* < 0.05 vs. T1DM + Chitosan, ‡ *p* < 0.05 vs. T1DM + Dapagliflozin, and & *p* < 0.05 vs. T1DM + Chitosan.

**Figure 5:**
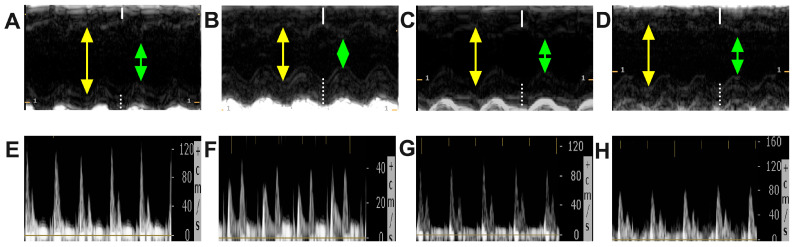
Representative images of M mode echocardiographic evaluation in mice without diabetes in the Control group (**A**), untreated mice in the T1DM group with diabetes (**B**), mice in the diabetic group treated with chitosan (T1MD + Chistosan) (**C**), and mice in the diabetic group treated with dapagliflozin (T1DM + Dapagliflozin) (**D**). E and A waves in mice without diabetes in the Control group (**E**), untreated mice in the T1DM group with diabetes (**F**), mice in the diabetic group treated with chitosan (T1MD + Chistosan) (**G**), and mice in the diabetic group treated with dapagliflozin (T1DM + Dapagliflozin) (**H**). The yellow arrows represent LV internal diastolic diameter (LVIDd), the green arrows represent LV internal systolic diameter (LVISd), the white solid lines represent interventricular septal width during end-diastole (IVSd), and the white dotted lines represent LV posterior wall width during end-diastole (VPWd).

An echocardiographic evaluation was performed both before the administration of streptozotocin, to determine all parameters at baseline, and then once every 4 weeks to analyze the evolution of these parameters. For all the animals included in our study, the ejection fraction of the left ventricle was calculated to assess the systolic function, as shown in Figure 3. We noticed that LVEF was a parameter that did not evolve much throughout our study (Figure 4A); approximately 8 weeks after the induction of diabetes, however, LVEF was lower in T1DM (65.81 ± 4.68%) and T1DM + Chitosan (70.43 ± 2.42%) compared to the Control group (75.21 ± 3.40%). This decrease remained steady until the end of the experiment (evaluation 12 weeks after the onset of diabetes) but was more pronounced in the T1DM group (to 55.11 ± 5.62%) compared to the control group (76.32 ± 2.84%). This decrease was much lower in the case of the groups treated with chitosan (63.41 ± 4.88%) and particularly with dapagliflozin (66.53 ± 4.08%). Moreover, reductions in left ventricle fractional shortening (LVFS), representing an improvement, were observed for chitosan and dapagliflozin (Figure 4B). Regarding the end-diastolic and end-systolic volumes (Figure 4C,D), although decreases in LVEDV and increases in LVESV were noticed, they were not found to be statistically significant different except in the case of LVESV, which was greatly increased in the T1DM and T1DM + Chitosan groups compared to the Control group (*p* < 0.05). Regarding the intracavitary diameters of the left ventricle, there was a decrease in the diastolic diameter and an increase in the systolic diameter even in the presence of diabetes, though no statistically significant differences were recorded (Figure 4E,F). It was observed, however, that the thickness of the interventricular septum (IVSd) and the posterior wall of the LV (LVPWd) were greater in the groups of animals with DM compared to those without DM 12 weeks after STZ administration, but left ventricular hypertrophy (representative images in Figure 5A–D) was improved by chitosan and particularly by dapagliflozin (Figure 4G,H). The analysis of diastolic profile (Figure 5E–H) highlighted a reduction in the amplitude of the E wave and a slight increase in the amplitude of the A wave in animals with DM, with these changes causing a decrease in the E/A ratio, thus fulfilling the definition of diastolic dysfunction. The treatment with chitosan, and particularly the treatment with dapagliflozin, prevented the occurrence of diastolic dysfunction 12 weeks after the onset of diabetes (Figure 4I–K). Finally, a reduction in stroke volume (SV) was observed in animals with DM, a decrease improved by 12 weeks of treatment with chitosan and especially with dapagliflozin (Figure 4L). The results of the ANOVA tests for each analyzed echocardiographic parameter can be found in Tables S6–S17.

### 2.5. Effect of Chitosan and Dapagliflozin on Cardiac Fibrosis

**Figure 6:**
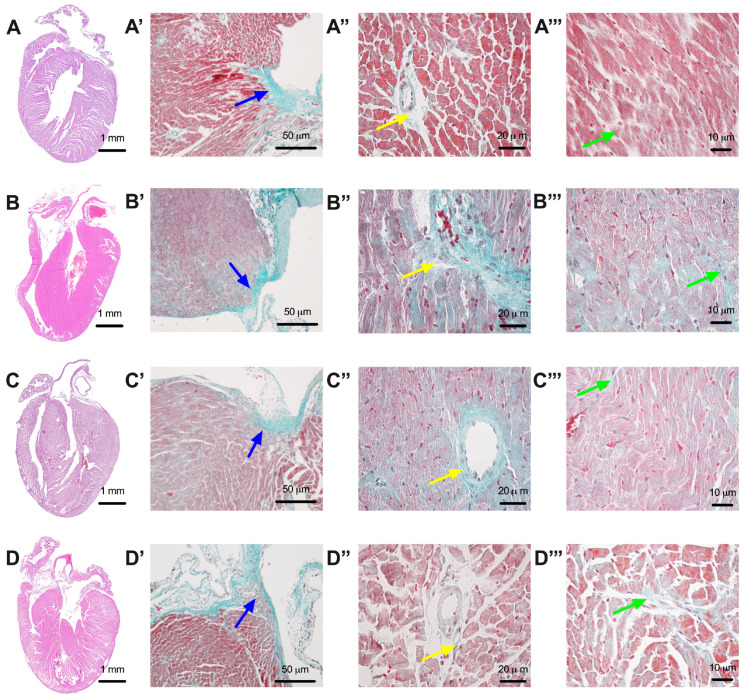
Representative images of mice hearts from the group of Control animals without diabetes (**A**), the group of untreated animals with diabetes ((**B**)—T1DM), the group of animals with diabetes that were treated with chitosan ((**C**)—T1DM + Chitosan), and the group of animals with diabetes that were treated with dapagliflozin ((**D**)—T1DM + Dapagliflozin). Capital letters with ’ indicate representative images of fibrosis at the atrioventricular junction (blue arrows). Capital letters with ” indicate representative images of perivascular fibrosis (yellow arrows). Capital letters with ’’’ indicate representative images of intramural myocardial fibrosis (green arrows).

**Figure 7:**
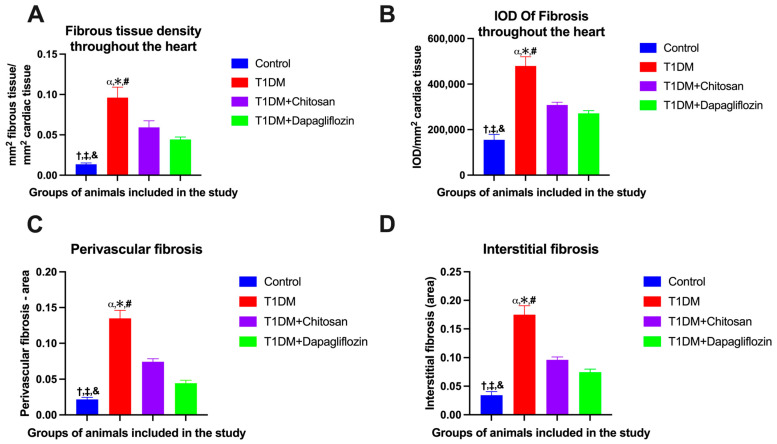
Effect of chitosan and dapagliflozin on cardiac fibrosis (mean ± S.E.M). (**A**) Fibrous tissue density at cardiac level (mm^2^ fibrous tissue/mm^2^ cardiac tissue). (**B**) Integrated optical density (IOD) reported per mm^2^ of cardiac tissue. (**C**) Perivascular fibrosis area. (**D**) Interstitial fibrosis area. † *p* < 0.05 vs. T1DM, ‡ *p* < 0.05 vs. T1DM + Chitosan, and & *p* < 0.05 vs. T1DM + Dapagliflozin, α *p* < 0.05 vs. Control, * *p* < 0.05 vs. T1DM + Chitosan, and # *p* < 0.05 vs. T1DM + Dapagliflozin.

Initially, all slices were stained with hematoxylin and eosin (Figure 6A–D) and later with Masson’s trichrome stain to highlight the collagen fibers and evaluate the degree of fibrosis. Each slice was initially scanned and then the area of fibrosis determined, which is related to the area of the respective slice. Fibrosis was analyzed considering the entire slice, including fibrosis at the level of the atrioventricular junction (Figure 6, capital letters with ’) and around the blood vessels (Figure 6, capital letters with ”) as well as intramural fibrosis (Figure 6, capital letters with ’’’). In mice without diabetes in the Control group, we observed the existence of fibrosis only around the blood vessels and at the atrioventricular junction, where the density of the fibrous tissue was 0.0135 ± 0.0017 mm^2^ fibrous tissue/mm^2^ cardiac tissue and IOD 155,010 ± 23,746. However, a significant increase in these parameters was observed in the mice from the T1DM group for which no therapy was administered, with the fibrous tissue density being 0.0961 ± 0.0130 mm^2^ fibrous tissue/mm^2^ cardiac tissue and IOD 479,543 ± 40,623. The degree of myocardial fibrosis was greatly suppressed by the administration of both chitosan (where the density determined from the fibrous test was 0.059 ± 0.0081 mm^2^ fibrous tissue/mm^2^ cardiac tissue and IOD 308,444 ± 12,050) and particularly by the administration of dapagliflozin (where the density determined from the fibrous test was 0.044 ± 0.0030 mm^2^ fibrous tissue/mm^2^ cardiac tissue and IOD 271,382 ± 11,780). These variations, as well as the statistical significance, are shown in Figure 7, and the results of the ANOVA test in Table S18 for the area of the fibrous tissue and in Table S19 for the integrated optical density (IOD) of the fibrous tissue. In addition to assessing the overall cross-sectional area for fibrosis, we examined both perivascular and interstitial fibrosis. Animals with T1DM exhibited significantly higher levels of perivascular and, notably, interstitial fibrosis compared to those without diabetes (*p* < 0.05, Figure 7C,D). Furthermore, the therapeutic effects of chitosan and dapagliflozin in animals with T1DM mitigated both perivascular and interstitial fibrosis”.

Moreover, the therapeutic effects exerted by chitosan and dapagliflozin in animals with T1DM attenuated both the degree of perivascular and interstitial fibrosis.

### 2.6. Effect of Chitosan and Dapaglifozin on Cardiac Oxidative Stress

**Figure 8:**
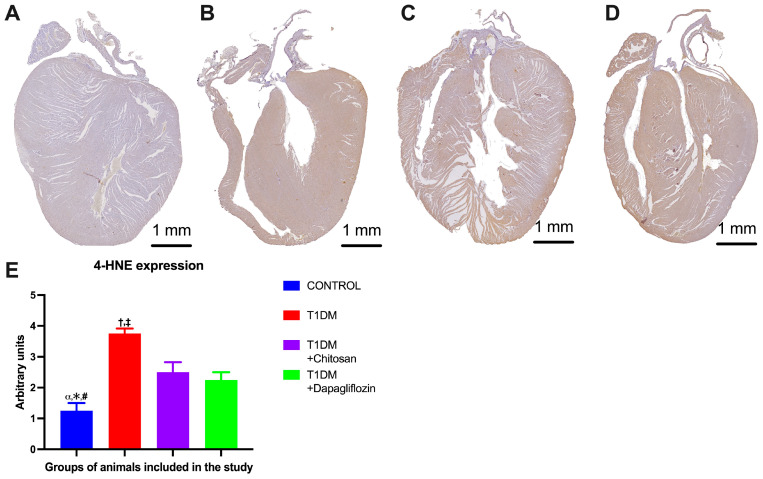
Representative images of 4-hydroxynonenal (HNE) immunoexpression in mice without diabetes in the Control group (**A**), untreated mice in the T1DM group with diabetes (**B**), mice in the diabetic group treated with chitosan (T1MD + Chistosan) (**C**), and mice in the diabetic group treated with dapagliflozin (T1DM + Dapagliflozin) (**D**). (**E**) HNE immunoexpression (mean ± S.E.M.) analysis. α *p* < 0.05 vs. T1DM, * *p* < 0.05 vs. T1DM + Chitosan, # *p* < 0.05 vs. T1DM + Dapagliflozin, † *p* < 0.05 vs. T1DM + Chitosan, and ‡ *p* < 0.05 vs. T1DM + Dapagliflozin.

To analyze the level of oxidative stress in the hearts of the animals included in our study, we evaluated the immunoexpression of oxidized lipid 4-hydroxynonenal (HNE) as shown in the images in Figure 8A–D. We observed a marked increase in HNE immunoexpression in untreated DM (T1DM) mice compared to those of the Control group (1.250 ± 0.250 vs. 3.750 ± 0.163 arbitrary units—a.u., *p* < 0.05, Figure 8E). Moreover, treatment with chitosan resulted in a similar reduction in HNE immunoexpression (2.500 ± 0.327 a.u.) as with dapagliflozin (2.250 ± 0.250 a.u.). The results of the ANOVA test are shown in Table S20.

## 3. Discussion

The results of our study indicate that cases of diabetic cardiomyopathy, the administration of chitosan has the same protective effects as dapagliflozin. The etiology of diabetic cardiomyopathy involves multiple factors and is not fully understood [19,20,21,22,23].

Firstly, STZ-induced Type 1 Diabetes Mellitus is widely used as a crucial model in diabetic research because it effectively mimics important characteristics of the human disease in animal subjects. STZ, a naturally derived chemical from Streptomyces achromogenes, demonstrates specific toxicity toward pancreatic beta cells. The specific mechanism behind this phenomenon can be primarily attributed to the role of the glucose transporter 2 (GLUT2) receptor in facilitating the entry of STZ into these cells [1,19]. When STZ is given, it causes DNA alkylation, which results in the death of beta cells and a subsequent significant decrease in insulin production [23]. This pathophysiological mechanism imitates the lack of insulin observed in T1DM, despite having a clearly different cause compared to the autoimmune-mediated destruction of beta cells seen in human T1DM [23].

Secondly, the STZ-induced T1DM model is particularly valued for its rapid induction and progression of diabetes, which also allows rapid evaluation of hyperglycemia effects on key organs [20]. However, unlike human T1DM, which is characterized by an autoimmune response leading to a gradual loss of beta cells, STZ-induced diabetes results from a chemically driven, acute cytotoxic process [23]. Consequently, while this model excellently delineates the consequences of insulin deficiency and offers a viable platform for testing insulin replacements and other therapeutic strategies, it does not fully encapsulate the autoimmune aspects of human T1DM [21]. Furthermore, the model’s dependence on the GLUT2 receptor means that it is most effective in rodent studies, as the expression of this receptor in human beta cells differs significantly from that in rodents [23]. This discrepancy calls for cautious interpretation of results and a measured approach when extrapolating findings to human diabetic pathology and treatment. Moreover, STZ can also induce toxicity in other organs such as the heart. In T1DM, there is an increased level of oxidative stress, a level determined not only by hyperglycemia but also by STZ toxicity.

These adjustments are mostly related to punctuation and phrasing to enhance clarity and readability. Overall, the text is well-structured and informative!

In our study, we objectified the appearance of diabetic cardiomyopathy by analyzing intramyocardial fibrosis, echocardiographic analysis of heart remodeling (determining the ejection fraction of the left ventricle, the intracavitary diameters, the thickness of the heart walls to evaluate the degree of left ventricular hypertrophy, cardiac volumes, and also the diastolic profile to highlight diastolic dysfunction), as well as based on the level of oxidative stress. Since it is known from the literature that treatment with chitosan influences the lipid profile, we performed a comparative analysis of chitosan with dapagliflozin on the cholesterol and triglycerides of the mice included in our study.

### 3.1. Cardiac Fibrosis

Cardiac fibrosis is defined according to the accumulation of extracellular matrix (ECM) proteins in the spaces between heart cells. The primary cellular agents responsible for this process are activated fibroblasts and myofibroblasts [24,25]. The precise mechanisms responsible for the progression of abnormal myocardial fibrosis in diabetic cardiomyopathy remain incompletely elucidated. However, it is believed to be caused by intricate interplay among multiple factors, including elevated blood glucose levels, oxidative stress, inflammation, and changes in cardiac metabolism [26]. The mechanisms underlying diabetic macrovasculopathy differ from those of diabetic cardiomyopathy due to variations in the smooth muscle cells of vessels [26]. Thus, the exact cause of the accentuated conversion of cardiomyocytes to fibroblasts through the endothelial to mesenchymal transition (EndMT) in diabetic cardiomyopathy is still unknown [24]. Nevertheless, in a study conducted on a diabetic mice animal model, it was reported that collagen deposition occurred as a result of the extracellular matrix (ECM)-synthetic program being activated by cardiac fibroblasts, without their conversion into myofibroblasts [27]. Cardiac fibroblasts residing in the heart are the main cells responsible for extracellular matrix (ECM) production in diabetic cardiomyopathy. There are three primary pathways that contribute to this process [24]. These pathways involve the direct activation of cardiac fibroblasts through transforming growth factor beta (TGF-β) and hyperglycemia as well as the accumulation of advanced glycation end-products (AGEs) under hyperglycemia stress [24]. The accumulation of AGEs promotes the crosslinking of collagen fibers, which in turn stimulates the activation of cardiac fibroblasts [28]. This can result in the formation of fibrosis and myocardium stiffness. Furthermore, changes in the way the heart metabolizes energy, such as through utilization of fatty acids instead of glucose through pathways involving adipokines and endothelin-1 (ET-1), as well as the activation of cardiac fibroblasts through neurohumoral pathways, can also play a role in the formation of abnormal myocardial fibrosis [29]. In our study, we observed an important increase in myocardial fibrosis as assessed by Masson’s trichrome staining, but this increase was much lower in the mice that received dapagliflozin. Moreover, chitosan reduced the extent of fibrosis but to a lesser degree than dapagliflozin. In a recent study, the administration of chitosan in the form of epicardial-implanted hydrogel reduced the degree of myocardial fibrosis in both nonischemic and ischemic hearts, and this effect was associated with an important reduction in hypertrophic stress [30]. Another suggested mechanism by which chitosan reduces myocardial fibrosis is via the reduction of transforming growth factor beta (TGF-β), which causes inflammatory macrophages to no longer be effective with consequent myofibroblast transdifferentiation and matrix synthesis via the Smad protein-dependent pathway [31]. The information regarding the use of dapagliflozin in diabetic cardiomyopathy to reduce fibrosis is much more consistent, and the main mechanism is represented by a reduction of blood glucose, as shown in all subsequent generated molecular mechanisms [16]. Moreover, dapagliflozin has proven efficacy on left ventricular remodeling demonstrated even in recent clinical trials [32,33,34,35].

### 3.2. Oxidative Stress

Oxidative stress (OS) refers to a condition characterized by an imbalance between the production of reactive oxygen species (ROS) and their elimination or neutralization by the body’s oxidative defense systems [36]. ROS, including superoxide and hydrogen peroxide, can react with nitric oxide (NO) to form peroxynitrite, which has a wide range of detrimental effects. These effects include reduced (NO) bioavailability, increased inflammation, mitochondrial dysfunction, and the promotion of tissue fibrosis and remodeling [37]. In our study, we showed that untreated diabetes (T1DM) is associated with increased oxidative stress within the heart tissue, as evidenced by higher levels of oxidized lipid (HNE) immunoexpression. However, treatment with both chitosan and dapagliflozin showed promising effects in reducing this oxidative stress, indicating their potential roles in protecting the heart from the damaging effects of oxidative stress in diabetes. Regarding dapagliflozin, Arow M et al. demonstrated in their recent study that dapagliflozin reduces oxidative stress both by reducing the blood glucose level and also through an independent mechanism involving reduced intracellular calcium overload [16]. Overall, clarity is lacking regarding the studies on chitosan, and the exact mechanism for the decrease in oxidative stress remains unknown. This reduction could be attributed to either the antioxidant properties of chitosan or its ability to decrease the production of reactive oxygen species (ROS) through other effects, such as enhanced cardiac and/or mitochondrial function [6].

### 3.3. Echocardiographic Parameters

In the mice with diabetes in our initial study, we observed a high degree of myocardial fibrosis, as a consequence of increased oxidative stress, along with an increase in the thickness of the walls of the left ventricle and later an alteration in diastolic function, which manifested as a reduction in the E wave and slight increase in the A wave amplitude, sometimes resulting in the inversion of the E/A ratio. Moreover, 12 weeks after the onset of diabetes, we even observed a slight decrease in the ejection fraction of the left ventricle. These changes were greatly delayed particularly by dapagliflozin treatment but also in mice treated with chitosan, where an improvement was observed in terms of these cardiac manifestations from diabetes. Through the effects of reducing cardiac fibrosis but also through controlling multiple signaling pathways, dapagliflozin has a definite effect in preventing the occurrence of cardiac remodeling from diabetes [38,39]. Regarding chitosan, studies on its use in diabetic cardiomyopathy are lacking, but its use in myocardial regeneration has been analyzed especially in models of ischemic cardiomyopathy [30,40,41,42].

### 3.4. Lipid Profile

It is well known that in diabetes, increased levels of circulating free fatty acids primarily accumulate as triglycerides in adipose tissue. However, accumulation of fat in organs other than visceral and subcutaneous fat cells leads to lipotoxicity. This condition causes the dysfunction of various cells and organs, including the liver, pancreatic β cells, skeletal muscles, and myocardium [43]. Moreover, dyslipidemia is a cause of ischemic heart disease independent of the presence of diabetes mellitus [43]. Although we did not use an animal model with dyslipidemia, we observed a slight increase in triglycerides in mice with diabetes. Regarding the treatments, the lipid profile was not influenced by the administration of dapagliflozin, while the administration of chitosan caused a decrease in both triglycerides and total cholesterol. The use of dapagliflozin resulted in minor changes in lipid levels with an unclear clinical significance [44], and several studies have demonstrated that the use of chitosan has a definite role in lowering total cholesterol and triglycerides. Chitosan decreases the expression of CCAAT enhancer-binding proteins α (C/EBPα) as well as of peroxisome proliferator-activated receptor γ (PPARγ), thus reducing lipid accumulation [45,46]. Additionally, a reduction in lipids occurs through the downregulation of 3-hydroxy-3-methylglutaryl-CoA reductase (HMGCR), sterol regulatory element-binding protein-1c (SREBP-1c), or acetyl-CoA carboxylase (ACC) [6].

### 3.5. The Link between Oxidative Stress and Cardiac Fibrosis

The production of reactive oxygen species (ROS) and the resulting oxidative stress have been specifically associated with the development of cardiac fibrosis and cardiomyopathy [47]. In physiological circumstances, a small quantity of oxygen is converted into reactive oxygen species (ROS) [48]. However, in individuals with diabetes, an excessive amount of ROS is produced. ROS can stimulate transcription factors like nuclear factor kappa-light-chain-enhancer of activated B cells (NF-κB), which subsequently control the transcription of various pro-inflammatory genes, including Tumor Necrosis Factor α (TNF-α), Transforming Growth Factor β1 (TGF-β1), and Interleukins (IL-1β, IL-6, IL-18) [47]. These genes are known to play a role in the development of heart failure (HF) associated with diabetes mellitus (DM) [49]. TNF-α stimulation induces collagen synthesis, whereas IL-1β promotes the pro-inflammatory fibroblast phenotype [50].

Multiple studies have demonstrated that oxidative stress triggers cardiac fibrosis by promoting the expression of TGF-β1 [51,52]. TGF-β1 plays a crucial role in the development of tissue fibrosis [47]. Angiotensin II, TGF-β1/SMAD signaling, and protein kinase C (PKC) activity are stimulated by hyperglycemia and hyperinsulinemia in fibroblasts [53,54]. These processes subsequently trigger the accumulation of collagen in the interstitial space and the formation of fibrous tissue, which is linked to an elevated expression of TGF-β1. TGF-β1 facilitates the conversion of myofibroblasts and enhances the maintenance of the extracellular matrix [52].

Chronic angiotensin II triggers oxidative stress, which is associated with a pro-fibrogenic phenotype in the heart. This leads to changes in the extracellular matrix by decreasing matrix metalloproteinases (MMPs) [55]. In addition, NF-κB upregulates the expression of intercellular adhesion molecule (ICAM-1) and vascular cell adhesion molecule 1 (VCAM-1) [56].

### 3.6. Limitations of the Study

A major limitation of our study is the lack of genomic and comprehensive proteomic analysis for clarifying the molecular mechanisms underlying our observations. For example, we did not analyze the expression of the profibrotic genes *Col1a1*, *Col1a2*, and *Col3a1* or of hypertrophic genes *Anp*, *Bnp*, and *Myh7*. Additionally, as this study involves an examination of an animal model, further investigations are required to translate these findings to human pathology. Furthermore, the quantification of cholesterol and triglycerides was conducted only once, at the end of the study. For the assessment of oxidative stress, we used 4-HNE as the sole marker. Thus, our study lacks a detailed molecular characterization of cardiac fibrosis and oxidation. Plasma ketone bodies and glucosuria were not measured in the animals included in our study. Another limitation of our study is represented by pharmacological anesthesia and its potential influence on cardiac parameters, such as LVEF. In the animal model study, significantly elevated doses of the examined molecules were administered, in contrast to clinical studies conducted on human participants.

## 4. Materials and Methods

This study was approved together with all related protocols by the Ethics Committee of the University of Medicine and Pharmacy in Craiova, Romania (no. 37/20 January, 2023). All experiments on animals were carried out in the Animal Facility of the University of Medicine and Pharmacy of Craiova and all the regulations in the field stipulated by the National Research Council (US) Committee in Guide for the Care and Use of Laboratory Animals [57] and by the European Council Directive (86/609/EEC) were respected. Throughout the experiments, the animals were housed in a clean, pathogen-free environment, with a temperature ranging 23–25 °C, relative humidity of 40–70%, a constant day–night rhythm, and ad libitum access to diet and water.

### 4.1. Reagents

Streptozotocin (STZ, ≥98% HPLC, Sigma-Aldrich, Munich, Germany) and also the trichrome staining kit (Masson’s trichrome staining kit, catalog number 100485) were purchased from Sigma-Aldrich. Rabbit polyclonal anti-4-hydroxynonenal (4-HNE) was purchased from Bioss (catalog number ABIN873270, Freiburg, Germany). HRP-conjugated secondary antibody (Vector Laboratories, Newark, CA, USA, ImmPRESS^®^ HRP Goat Anti-Rabbit IgG Polymer Detection Kit, Peroxidase—MP-7451) was also used. 3,3′-Diaminobenzidine (DAB) was purchased from DAKO (Glostrup, Denmark). Medium molecular weight chitosan was purchased from Sigma-Aldrich (Catalog number 448877, Sigma-Aldrich Chemie GmbH, Taufkirchen, Germany).

### 4.2. Animals

In our study, we used male C57Bl/6 mice aged between 8 and 10 weeks. In the end, we used 32 animals randomly divided into 4 groups:

1. Control (without diabetes);
2. T1DM (type 1 diabetes mellitus);
3. T1DM + Chitosan (type 1 diabetes mellitus and treatment with chitosan);
4. T1DM + Dapagliflozin (type 1 diabetes mellitus and treatment with dapagliflozin).

It should be mentioned that each group initially contained 8–10 animals, taking into account that we had a mortality rate of approximately 10% (resulting especially from the toxicity due to the administration of streptozotocin, but we also had one animal that was killed by intragroup cannibalism, probably through polyphagia induced by diabetes).

Regarding induction and assessment of diabetes mellitus and therapeutic protocols in mice, diabetes was induced by a single intraperitoneal injection and the administration of 150 mg streptozotocin/kg body weight, between 8 and 10 weeks of life, as in two of our previous studies [58,59]. Diabetes mellitus was diagnosed according to the criteria of the American Diabetes Association (ADA) [60]. The mice in the T1DM + Chitosan and T1DM + Dapagliflozin groups received treatment for a period of 12 weeks after the induction of diabetes via gastric gavage. The chitosan dose was 150 mg/kg body weight [61] and the dapagliflozin dose was 10 mg/kg body weight [59]. The blood glucose level was determined both before STZ administration once every 2 weeks to monitor the onset and evolution of diabetes. The level of fasting glucose in the blood of animals was determined by taking the blood after leaving the animals without food in the afternoon for 3–4 h prior to blood sampling. The determination was made by means of a standard glucometer (Contour Plus One, Ascensia Diabetes Care, Basel, Switzerland) based on blood taken from a large vein located in the tail of the animal. Total cholesterol and triglycerides were determined only once, at the end of the experiment, by means of a standard analyzer (MulticareIN, Biochemical Systems International, Milan, Italy) with blood taken when the animal was sacrificed by puncturing the inferior vena cava.

### 4.3. Echocardiography

The echocardiographic examination was performed before enrolling in the study and subsequently every 4 weeks, with 4 examinations being performed until the end of the study. A Philips CX50 (Netherlands) ultrasound machine equipped with a high-resolution L12-3 linear array transducer (frequency range 12–3 MHz) and software (5.5.3. version) for cardiovascular applications was used. For the echocardiographic evaluation, each animal was anesthetized with a cocktail of ketamine (50 mg/mL)/xylazine (7 mg/mL) and then placed on a homothermic platform (37 °C). Standard sections were used as recommended by professional societies in this field [62]. During the examination in parasternal long axis view, left ventricle (LV) end-diastolic volume (LVEDV), LV end-systolic volume (LVESV), and stroke volume (SV) were determined. By relating the stroke volume to the LVEDV and multiplying by 100, we obtained the ejection fraction of the left ventricle (LVEF). During the M mode examination, we determined LV internal diastolic diameter (LVIDd), LV internal systolic diameter (LVISd), IVSd interventricular septal width during end-diastole (IVSd), LV posterior wall width during end-diastole (LVPWd), and fractional shortening (FS). Pulsed Doppler echocardiography at the mitral inflow level was used to measure the E wave (early diastole), A wave (atrial systole), and E/A ratio. Echocardiography and assessments were performed in blind conditions.

### 4.4. Histology and Immunohistochemistry Assessment

At the end of the experiment, all animals were euthanized by injecting a high dose of ketamine/xylazine. The heart was sampled and immediately immersed in a potassium chloride solution to induce diastolic arrest. Later, the heart was fixed in a 10% formalin solution in paraffin, and 4 micrometer thick sections were prepared using a microtome (HM355S). These were placed on slides with poly-L-lysine and then deparaffinized using the dewatering method. Initially, a slice from each tissue block was stained with hematoxylin and eosin. Then, another set of slices was stained using Masson’s trichrome staining method to assess the degree of fibrosis. To evaluate oxidative stress, the expression of HNE (4-hydroxynonenal) was determined. The slices were immunostained at 4 °C overnight with primary antibody (anti HNE). Then, the slices were incubated with HRP-conjugated secondary antibody. The primary antibody was detected using a peroxidase-based kit and visualized using 3,3′-diaminobenzidine (DAB) substrate with enhancer. Then, the slides were subsequently counterstained with hematoxylin (DAKO). The slices were digitized by scanning with a microscope with a MoticEasyScan Pro 6 scanner (Kowloon, Hong Kong) using a 20× objective. EasyScanner software version 6 and Image ProPlus AMS 9 software (version 9, Media Cybernetics, Rockville, MD, USA) were used for imaging analysis. The total cardiac tissue area (mm^2^) was calculated for each slice, and for the color signal, both the area and the integrated optical density (IOD) were calculated. The signal area as well as the IOD were calculated in mm^2^.

### 4.5. Statistical Analysis

All results are expressed as mean and standard deviation (S.D.) or the standard error of the mean (S.E.M.), and the threshold of *p* < 0.05 was used to establish statistical significance. All data were analyzed with GraphPad Prism software (Version 10.0, San Diego, CA, USA). In cases where there were more than two groups of data, we analyzed the difference between the groups by means of the ANOVA variant test.

## 5. Conclusions

The results of our study demonstrate that chitosan exhibits promising effects in the treatment of diabetic cardiomyopathy that are comparable with the beneficial effects, already demonstrated, of dapagliflozin, namely in reducing the degree of intramyocardial fibrosis and ameliorating the deterioration of echocardiographic parameters.

## Data Availability

Data are contained within the article or available upon request from the corresponding authors.

## Supplementary Materials

The following supporting information can be downloaded at https://www.mdpi.com/article/10.3390/ijms25042118/s1.
