# Supplementary material for: Chitosan Versus Dapagliflozin in a Diabetic Cardiomyopathy Mouse Model
Source: Int J Mol Sci. 2024 Feb 9;25(4):2118. doi: 10.3390/ijms25042118 (PMC10888683; doi:10.3390/ijms25042118)
Supplement: Supplementary file 1 [file ijms-25-02118-s001.zip › ijms-2841502-supplementary.pdf]

Supplementary Materials

Table S1. Plasma glucose level (mean and SD).

	Control		T1DM		T1DM+Chitosan		T1DM+Dapagliflozin	
Weeks	Mean	SD	Mean	SD	Mean	SD	Mean	SD
8	105,38	16,69	107,75	25,56	113,38	17,53	111,88	16,17
11	125,00	28,54	548,00	39,26	483,88	81,17	417,25	61,21
14	121,75	36,38	546,25	51,14	529,50	47,66	437,75	79,81
17	119,50	35,80	556,88	45,24	568,50	28,87	421,25	68,76
20	120,63	27,62	574,50	30,56	537,88	38,31	426,00	49,34

Table S2. ANOVA test results on plasma glucose levels.

Tukey's multiple comparisons test	Adjusted P Value	Summary
Week 8 (before STZ)		
Control vs. T1DM	0,9828	ns
Control vs. T1DM+Chitosan	0,7481	ns
Control vs. T1DM+Dapagliflozin	0,8477	ns
T1DM vs. T1DM+Chitosan	0,9417	ns
T1DM vs. T1DM+Dapagliflozin	0,9751	ns
T1DM+Chitosan vs. T1DM+Dapagliflozin	0,7545	ns
Week 11 (after STZ)		
Control vs. T1DM	<0,0001	****
Control vs. T1DM+Chitosan	<0,0001	****
Control vs. T1DM+Dapagliflozin	<0,0001	****
T1DM vs. T1DM+Chitosan	0,2952	ns
T1DM vs. T1DM+Dapagliflozin	0,0207	*
T1DM+Chitosan vs. T1DM+Dapagliflozin	0,4055	ns
Week 14 (after STZ)		
Control vs. T1DM	<0,0001	****
Control vs. T1DM+Chitosan	<0,0001	****
Control vs. T1DM+Dapagliflozin	<0,0001	****
T1DM vs. T1DM+Chitosan	0,9187	ns
T1DM vs. T1DM+Dapagliflozin	0,1009	ns
T1DM+Chitosan vs. T1DM+Dapagliflozin	0,1749	ns
Week 17 (after STZ)		
Control vs. T1DM	<0,0001	****
Control vs. T1DM+Chitosan	<0,0001	****
Control vs. T1DM+Dapagliflozin	<0,0001	****
T1DM vs. T1DM+Chitosan	0,905	ns
T1DM vs. T1DM+Dapagliflozin	0,0162	*
T1DM+Chitosan vs. T1DM+Dapagliflozin	0,0045	**
Week 20 (after STZ)		
Control vs. T1DM	<0,0001	****
Control vs. T1DM+Chitosan	<0,0001	****
Control vs. T1DM+Dapagliflozin	<0,0001	****
T1DM vs. T1DM+Chitosan	0,3322	ns
T1DM vs. T1DM+Dapagliflozin	0,0015	**
T1DM+Chitosan vs. T1DM+Dapagliflozin	0,0001	***

Table S3. Weight of study animals at enrollment.

	Control	T1DM	T1DM+Chitosan	T1DM+Dapagliflozin
Minimum	22,90	23,30	23,90	23,10
25% Percei	25,33	24,30	24,83	24,25
Median	26,10	26,40	26,50	25,40
75% Percei	26,88	27,70	27,73	26,68
Maximum	27,50	28,20	28,10	27,50
Mean	25,90	26,09	26,33	25,41
Std. Deviati	1,41	1,86	1,53	1,49

Table S4. ANOVA test results on total cholesterol (TC) concentration after treatment for 12 weeks.

Tukey's multiple comparisons test	Adjusted P Value	Summary
Control vs. T1DM	0,6045	ns
Control vs. T1DM+Chitosan	<0,0001	****
Control vs. T1DM+Dapagliflozin	0,9913	ns
T1DM vs. T1DM+Chitosan	<0,0001	****
T1DM vs. T1DM+Dapagliflozin	0,776	ns
T1DM+Chitosan vs. T1DM+Dapaglifloz	<0,0001	****

Table S5. ANOVA test results on triglycerides concentration after treatment for 12 weeks.

Tukey's multiple comparisons test	Adjusted P Value	Summary
Control vs. T1DM	<0,0001	****
Control vs. T1DM+Chitosan	0,0082	**
Control vs. T1DM+Dapagliflozin	0,0006	***
T1DM vs. T1DM+Chitosan	<0,0001	****
T1DM vs. T1DM+Dapagliflozin	0,1143	ns
T1DM+Chitosan vs. T1DM+Dapagliflozin	<0,0001	****

Table S6. ANOVA test results on left ventricular ejection fraction (LVEF).

Tukey's multiple comparisons test	Adjusted P Value	Summary
Row 2		
Control vs. T1DM	0,9281	ns
Control vs. T1DM+Chitosan	0,4072	ns
Control vs. T1DM+Dapagliflozin	0,8174	ns
T1DM vs. T1DM+Chitosan	0,7506	ns
T1DM vs. T1DM+Dapagliflozin	0,9912	ns
T1DM+Chitosan vs. T1DM+Dapaglifl	0,9078	ns
Row 3		
Control vs. T1DM	0,0666	ns
Control vs. T1DM+Chitosan	0,3512	ns
Control vs. T1DM+Dapagliflozin	0,2175	ns
T1DM vs. T1DM+Chitosan	0,4158	ns
T1DM vs. T1DM+Dapagliflozin	0,8664	ns
T1DM+Chitosan vs. T1DM+Dapaglifl	0,8768	ns
Row 4		
Control vs. T1DM	0,0026	**
Control vs. T1DM+Chitosan	0,0298	*
Control vs. T1DM+Dapagliflozin	0,3789	ns
T1DM vs. T1DM+Chitosan	0,1218	ns
T1DM vs. T1DM+Dapagliflozin	0,081	ns
T1DM+Chitosan vs. T1DM+Dapaglifl	0,8522	ns
Row 5		
Control vs. T1DM	<0,0001	****
Control vs. T1DM+Chitosan	0,0002	***
Control vs. T1DM+Dapagliflozin	0,0005	***
T1DM vs. T1DM+Chitosan	0,0323	*
T1DM vs. T1DM+Dapagliflozin	0,0023	**
T1DM+Chitosan vs. T1DM+Dapaglifl	0,5285	ns

Table S7. ANOVA test results on fractional shortening (FS).

Tukey's multiple comparisons t	Adjusted P Value	Summary
Row 2		
Control vs. T1DM	0,7629	ns
Control vs. T1DM+Chitosan	0,9998	ns
Control vs. T1DM+Dapaglifozi	0,7021	ns
T1DM vs. T1DM+Chitosan	0,8916	ns
T1DM vs. T1DM+Dapagliflozin	0,9977	ns
T1DM+Chitosan vs. T1DM+Da	0,8553	ns
Row 3		
Control vs. T1DM	0,4114	ns
Control vs. T1DM+Chitosan	0,959	ns
Control vs. T1DM+Dapaglifozi	0,1762	ns
T1DM vs. T1DM+Chitosan	0,5238	ns
T1DM vs. T1DM+Dapagliflozin	0,77	ns
T1DM+Chitosan vs. T1DM+Da	0,214	ns
Row 4		
Control vs. T1DM	0,022	*
Control vs. T1DM+Chitosan	0,0565	ns
Control vs. T1DM+Dapaglifozi	0,1723	ns
T1DM vs. T1DM+Chitosan	0,9754	ns
T1DM vs. T1DM+Dapagliflozin	0,842	ns
T1DM+Chitosan vs. T1DM+Da	0,9721	ns
Row 5		
Control vs. T1DM	<0,0001	****
Control vs. T1DM+Chitosan	0,0014	**
Control vs. T1DM+Dapaglifozi	0,0014	**
T1DM vs. T1DM+Chitosan	0,0331	*
T1DM vs. T1DM+Dapagliflozin	0,1109	ns
T1DM+Chitosan vs. T1DM+Da	0,9959	ns

Table S8. ANOVA test results on left ventricle (LV) end-diastolic volume (LVEDV).

Tukey's multiple comparisons test	Adjusted P Value	Summary
Row 2		
Control vs. T1DM	0,9993	ns
Control vs. T1DM+Chitosan	0,9852	ns
Control vs. T1DM+Dapagliflozin	0,8388	ns
T1DM vs. T1DM+Chitosan	0,9677	ns
T1DM vs. T1DM+Dapagliflozin	0,7623	ns
T1DM+Chitosan vs. T1DM+Dapagliflozin	0,9838	ns
Row 3		
Control vs. T1DM	0,9757	ns
Control vs. T1DM+Chitosan	0,9824	ns
Control vs. T1DM+Dapagliflozin	0,6416	ns
T1DM vs. T1DM+Chitosan	>0,9999	ns
T1DM vs. T1DM+Dapagliflozin	0,8692	ns
T1DM+Chitosan vs. T1DM+Dapagliflozin	0,9383	ns
Row 4		
Control vs. T1DM	0,7873	ns
Control vs. T1DM+Chitosan	0,8183	ns
Control vs. T1DM+Dapagliflozin	0,5001	ns
T1DM vs. T1DM+Chitosan	0,998	ns
T1DM vs. T1DM+Dapagliflozin	0,9365	ns
T1DM+Chitosan vs. T1DM+Dapagliflozin	0,9911	ns
Row 5		
Control vs. T1DM	0,1871	ns
Control vs. T1DM+Chitosan	0,2371	ns
Control vs. T1DM+Dapagliflozin	0,1232	ns
T1DM vs. T1DM+Chitosan	0,9961	ns
T1DM vs. T1DM+Dapagliflozin	0,9516	ns
T1DM+Chitosan vs. T1DM+Dapagliflozin	0,9941	ns

Table S9. ANOVA test results on LV end-systolic volume (LVESV).

Tukey's multiple comparisons test	Adjusted P Value	Summary
Row 2		
Control vs. T1DM	0,9047	ns
Control vs. T1DM+Chitosan	0,7612	ns
Control vs. T1DM+Dapagliflozin	0,9875	ns
T1DM vs. T1DM+Chitosan	0,9746	ns
T1DM vs. T1DM+Dapagliflozin	0,995	ns
T1DM+Chitosan vs. T1DM+Dapagliflozin	0,9361	ns
Row 3		
Control vs. T1DM	0,2282	ns
Control vs. T1DM+Chitosan	0,8739	ns
Control vs. T1DM+Dapagliflozin	0,8756	ns
T1DM vs. T1DM+Chitosan	0,7956	ns
T1DM vs. T1DM+Dapagliflozin	0,7827	ns
T1DM+Chitosan vs. T1DM+Dapagliflozin	>0,9999	ns
Row 4		
Control vs. T1DM	0,0545	ns
Control vs. T1DM+Chitosan	0,7763	ns
Control vs. T1DM+Dapagliflozin	0,9895	ns
T1DM vs. T1DM+Chitosan	0,5118	ns
T1DM vs. T1DM+Dapagliflozin	0,1875	ns
T1DM+Chitosan vs. T1DM+Dapagliflozin	0,9333	ns
Row 5		
Control vs. T1DM	<0,0001	****
Control vs. T1DM+Chitosan	0,048	*
Control vs. T1DM+Dapagliflozin	0,23	ns
T1DM vs. T1DM+Chitosan	0,062	ns
T1DM vs. T1DM+Dapagliflozin	0,0026	**
T1DM+Chitosan vs. T1DM+Dapagliflozin	0,6556	ns

Table S10. ANOVA test results on LV internal diastolic diameter (LVIDd).

Tukey's multiple comparisons test	Adjusted P Value	Summary
Row 2		
Control vs. T1DM	0,3962	ns
Control vs. T1DM+Chitosan	0,3036	ns
Control vs. T1DM+Dapagliflozin	0,7593	ns
T1DM vs. T1DM+Chitosan	0,9674	ns
T1DM vs. T1DM+Dapagliflozin	0,9449	ns
T1DM+Chitosan vs. T1DM+Dapagliflozin	0,7958	ns
Row 3		
Control vs. T1DM	0,2315	ns
Control vs. T1DM+Chitosan	0,2119	ns
Control vs. T1DM+Dapagliflozin	0,8647	ns
T1DM vs. T1DM+Chitosan	0,9434	ns
T1DM vs. T1DM+Dapagliflozin	0,6602	ns
T1DM+Chitosan vs. T1DM+Dapagliflozin	0,5007	ns
Row 4		
Control vs. T1DM	0,4623	ns
Control vs. T1DM+Chitosan	0,4492	ns
Control vs. T1DM+Dapagliflozin	0,9135	ns
T1DM vs. T1DM+Chitosan	0,9802	ns
T1DM vs. T1DM+Dapagliflozin	0,8182	ns
T1DM+Chitosan vs. T1DM+Dapagliflozin	0,7344	ns
Row 5		
Control vs. T1DM	0,9979	ns
Control vs. T1DM+Chitosan	0,814	ns
Control vs. T1DM+Dapagliflozin	0,9859	ns
T1DM vs. T1DM+Chitosan	0,7373	ns
T1DM vs. T1DM+Dapagliflozin	0,9982	ns
T1DM+Chitosan vs. T1DM+Dapagliflozin	0,6684	ns

Table S11. ANOVA test results on LV internal systolic diameter (LVISd).

Tukey's multiple comparisons test	Adjusted P Value	Summary
Row 2		
Control vs. T1DM	0,3102	ns
Control vs. T1DM+Chitosan	0,5183	ns
Control vs. T1DM+Dapagliflozin	0,6327	ns
T1DM vs. T1DM+Chitosan	0,9995	ns
T1DM vs. T1DM+Dapagliflozin	0,9835	ns
T1DM+Chitosan vs. T1DM+Dapagliflozin	0,9814	ns
Row 3		
Control vs. T1DM	0,1432	ns
Control vs. T1DM+Chitosan	0,168	ns
Control vs. T1DM+Dapagliflozin	0,428	ns
T1DM vs. T1DM+Chitosan	>0,9999	ns
T1DM vs. T1DM+Dapagliflozin	0,9441	ns
T1DM+Chitosan vs. T1DM+Dapagliflozin	0,9567	ns
Row 4		
Control vs. T1DM	0,0443	*
Control vs. T1DM+Chitosan	0,0747	ns
Control vs. T1DM+Dapagliflozin	0,4306	ns
T1DM vs. T1DM+Chitosan	0,9966	ns
T1DM vs. T1DM+Dapagliflozin	0,7636	ns
T1DM+Chitosan vs. T1DM+Dapagliflozin	0,7293	ns
Row 5		
Control vs. T1DM	0,0793	ns
Control vs. T1DM+Chitosan	0,1031	ns
Control vs. T1DM+Dapagliflozin	0,4633	ns
T1DM vs. T1DM+Chitosan	0,9917	ns
T1DM vs. T1DM+Dapagliflozin	0,8047	ns
T1DM+Chitosan vs. T1DM+Dapagliflozin	0,729	ns

Table S12. ANOVA test results on interventricular septal width during end-diastole (IVSd).

Row 2		
Control vs. T1DM	0,6323	ns
Control vs. T1DM+Chitosan	0,9988	ns
Control vs. T1DM+Dapagliflozin	0,9993	ns
T1DM vs. T1DM+Chitosan	0,7601	ns
T1DM vs. T1DM+Dapagliflozin	0,6809	ns
T1DM+Chitosan vs. T1DM+Dapagliflozin	>0,9999	ns
Row 3		
Control vs. T1DM	0,7655	ns
Control vs. T1DM+Chitosan	0,6671	ns
Control vs. T1DM+Dapagliflozin	>0,9999	ns
T1DM vs. T1DM+Chitosan	0,9903	ns
T1DM vs. T1DM+Dapagliflozin	0,735	ns
T1DM+Chitosan vs. T1DM+Dapagliflozin	0,6346	ns
Row 4		
Control vs. T1DM	0,0161	*
Control vs. T1DM+Chitosan	0,3116	ns
Control vs. T1DM+Dapagliflozin	0,9857	ns
T1DM vs. T1DM+Chitosan	0,2095	ns
T1DM vs. T1DM+Dapagliflozin	0,0099	**
T1DM+Chitosan vs. T1DM+Dapagliflozin	0,194	ns
Row 5		
Control vs. T1DM	<0,0001	****
Control vs. T1DM+Chitosan	<0,0001	****
Control vs. T1DM+Dapagliflozin	0,0865	ns
T1DM vs. T1DM+Chitosan	0,1395	ns
T1DM vs. T1DM+Dapagliflozin	0,0119	*
T1DM+Chitosan vs. T1DM+Dapagliflozin	0,2331	ns

Table S13. ANOVA test results on LV posterior wall width during end-diastole (LVPWd).

Tukey's multiple comparisons test	Adjusted P Value	Summary
Row 2		
Control vs. T1DM	0,9038	ns
Control vs. T1DM+Chitosan	0,9995	ns
Control vs. T1DM+Dapagliflozin	>0,9999	ns
T1DM vs. T1DM+Chitosan	0,9571	ns
T1DM vs. T1DM+Dapagliflozin	0,9006	ns
T1DM+Chitosan vs. T1DM+Dapaglifl	0,9998	ns
Row 3		
Control vs. T1DM	0,9438	ns
Control vs. T1DM+Chitosan	0,9955	ns
Control vs. T1DM+Dapagliflozin	0,996	ns
T1DM vs. T1DM+Chitosan	0,9755	ns
T1DM vs. T1DM+Dapagliflozin	0,9792	ns
T1DM+Chitosan vs. T1DM+Dapaglifl	>0,9999	ns
Row 4		
Control vs. T1DM	0,0565	ns
Control vs. T1DM+Chitosan	0,502	ns
Control vs. T1DM+Dapagliflozin	0,9984	ns
T1DM vs. T1DM+Chitosan	0,289	ns
T1DM vs. T1DM+Dapagliflozin	0,037	*
T1DM+Chitosan vs. T1DM+Dapaglifl	0,3338	ns
Row 5		
Control vs. T1DM	<0,0001	****
Control vs. T1DM+Chitosan	0,008	**
Control vs. T1DM+Dapagliflozin	0,0723	ns
T1DM vs. T1DM+Chitosan	0,0539	ns
T1DM vs. T1DM+Dapagliflozin	0,0135	*
T1DM+Chitosan vs. T1DM+Dapaglifl	0,8244	ns

Table S14. ANOVA test results on E wave (early diastole).

Tukey's multiple comparisons test	Adjusted P Value	Summary
Row 2		
Control vs. T1DM	0,9967	ns
Control vs. T1DM+Chitosan	0,9138	ns
Control vs. T1DM+Dapagliflozin	0,6687	ns
T1DM vs. T1DM+Chitosan	0,9587	ns
T1DM vs. T1DM+Dapagliflozin	0,7009	ns
T1DM+Chitosan vs. T1DM+Dapagliflozin	0,8499	ns
Row 3		
Control vs. T1DM	0,4558	ns
Control vs. T1DM+Chitosan	0,4273	ns
Control vs. T1DM+Dapagliflozin	0,1345	ns
T1DM vs. T1DM+Chitosan	>0,9999	ns
T1DM vs. T1DM+Dapagliflozin	0,6379	ns
T1DM+Chitosan vs. T1DM+Dapagliflozin	0,6352	ns
Row 4		
Control vs. T1DM	0,0601	ns
Control vs. T1DM+Chitosan	0,1007	ns
Control vs. T1DM+Dapagliflozin	0,0678	ns
T1DM vs. T1DM+Chitosan	0,9688	ns
T1DM vs. T1DM+Dapagliflozin	0,9962	ns
T1DM+Chitosan vs. T1DM+Dapagliflozin	0,9907	ns
Row 5		
Control vs. T1DM	<0,0001	****
Control vs. T1DM+Chitosan	0,0001	***
Control vs. T1DM+Dapagliflozin	0,0019	**
T1DM vs. T1DM+Chitosan	0,002	**
T1DM vs. T1DM+Dapagliflozin	<0,0001	****
T1DM+Chitosan vs. T1DM+Dapagliflozin	0,0568	ns

Table S15. ANOVA test results on A wave (atrial systole).

Tukey's multiple comparisons test	Adjusted P Value	Summary
Row 2		
Control vs. T1DM	0,9994	ns
Control vs. T1DM+Chitosan	0,9784	ns
Control vs. T1DM+Dapagliflozin	0,922	ns
T1DM vs. T1DM+Chitosan	0,9802	ns
T1DM vs. T1DM+Dapagliflozin	0,9576	ns
T1DM+Chitosan vs. T1DM+Dapagliflo	0,9998	ns
Row 3		
Control vs. T1DM	0,6495	ns
Control vs. T1DM+Chitosan	0,8661	ns
Control vs. T1DM+Dapagliflozin	0,5905	ns
T1DM vs. T1DM+Chitosan	0,9436	ns
T1DM vs. T1DM+Dapagliflozin	0,9896	ns
T1DM+Chitosan vs. T1DM+Dapagliflo	0,9827	ns
Row 4		
Control vs. T1DM	0,0057	**
Control vs. T1DM+Chitosan	0,0004	***
Control vs. T1DM+Dapagliflozin	0,0448	*
T1DM vs. T1DM+Chitosan	0,995	ns
T1DM vs. T1DM+Dapagliflozin	0,1963	ns
T1DM+Chitosan vs. T1DM+Dapagliflo	0,0208	*
Row 5		
Control vs. T1DM	0,0244	*
Control vs. T1DM+Chitosan	0,0008	***
Control vs. T1DM+Dapagliflozin	0,1143	ns
T1DM vs. T1DM+Chitosan	0,9984	ns
T1DM vs. T1DM+Dapagliflozin	0,3453	ns
T1DM+Chitosan vs. T1DM+Dapagliflo	0,0646	ns

Table S16. ANOVA test results on E/A ratio.

Row 2		
Control vs. T1DM	0,9997	ns
Control vs. T1DM+Chitosan	0,999	ns
Control vs. T1DM+Dapagliflozin	0,9854	ns
T1DM vs. T1DM+Chitosan	0,9951	ns
T1DM vs. T1DM+Dapagliflozin	0,992	ns
T1DM+Chitosan vs. T1DM+Dapagliflozin	0,9679	ns
Row 3		
Control vs. T1DM	0,1879	ns
Control vs. T1DM+Chitosan	0,3903	ns
Control vs. T1DM+Dapagliflozin	0,1092	ns
T1DM vs. T1DM+Chitosan	0,9318	ns
T1DM vs. T1DM+Dapagliflozin	0,9522	ns
T1DM+Chitosan vs. T1DM+Dapagliflozin	0,7173	ns
Row 4		
Control vs. T1DM	0,0041	**
Control vs. T1DM+Chitosan	0,0054	**
Control vs. T1DM+Dapagliflozin	0,019	*
T1DM vs. T1DM+Chitosan	0,991	ns
T1DM vs. T1DM+Dapagliflozin	0,176	ns
T1DM+Chitosan vs. T1DM+Dapagliflozin	0,1382	ns
Row 5		
Control vs. T1DM	<0,0001	****
Control vs. T1DM+Chitosan	0,0001	***
Control vs. T1DM+Dapagliflozin	0,0018	**
T1DM vs. T1DM+Chitosan	0,0015	**
T1DM vs. T1DM+Dapagliflozin	<0,0001	****
T1DM+Chitosan vs. T1DM+Dapagliflozin	0,0048	**

Table S17. ANOVA test results on stroke volume (SV).

Tukey's multiple comparisons test	Adjusted P Value	Summary
Row 2		
Control vs. T1DM	0,9983	ns
Control vs. T1DM+Chitosan	0,7945	ns
Control vs. T1DM+Dapagliflozin	0,6307	ns
T1DM vs. T1DM+Chitosan	0,814	ns
T1DM vs. T1DM+Dapagliflozin	0,6191	ns
T1DM+Chitosan vs. T1DM+Dapagliflozin	0,9972	ns
Row 3		
Control vs. T1DM	0,5049	ns
Control vs. T1DM+Chitosan	0,7838	ns
Control vs. T1DM+Dapagliflozin	0,1602	ns
T1DM vs. T1DM+Chitosan	0,9815	ns
T1DM vs. T1DM+Dapagliflozin	0,9649	ns
T1DM+Chitosan vs. T1DM+Dapagliflozin	0,8231	ns
Row 4		
Control vs. T1DM	0,0625	ns
Control vs. T1DM+Chitosan	0,2886	ns
Control vs. T1DM+Dapagliflozin	0,132	ns
T1DM vs. T1DM+Chitosan	0,9472	ns
T1DM vs. T1DM+Dapagliflozin	0,9443	ns
T1DM+Chitosan vs. T1DM+Dapagliflozin	0,9998	ns
Row 5		
Control vs. T1DM	<0,0001	****
Control vs. T1DM+Chitosan	0,0055	**
Control vs. T1DM+Dapagliflozin	0,0049	**
T1DM vs. T1DM+Chitosan	0,6172	ns
T1DM vs. T1DM+Dapagliflozin	0,4037	ns
T1DM+Chitosan vs. T1DM+Dapagliflozin	0,9936	ns

Table S 18. ANOVA test results on area of fibrosis.

Tukey's multiple comparisons test	Adjusted P Value	Summary
Control vs. T1DM	<0,0001	****
Control vs. T1DM+Chitosan	0,0017	**
Control vs. T1DM+Dapagliflozin	0,0472	*
T1DM vs. T1DM+Chitosan	0,013	*
T1DM vs. T1DM+Dapagliflozin	0,0004	***
T1DM+Chitosan vs. T1DM+Dapagliflozin	0,538	ns

Table S 19. ANOVA test results on IOD of fibrosis.

Tukey's multiple comparisons test	Adjusted P Value	Summary
Control vs. T1DM	<0,0001	****
Control vs. T1DM+Chitosan	0,0009	***
Control vs. T1DM+Dapagliflozin	0,0135	*
T1DM vs. T1DM+Chitosan	0,0002	***
T1DM vs. T1DM+Dapagliflozin	<0,0001	****
T1DM+Chitosan vs. T1DM+Dapagliflozin	0,7227	ns

Table S20. ANOVA test results on 4-HNE immunoexpression.

Tukey's multiple comparisons test	Adjusted P Value	Summary
CONTROL vs. T1DM	<0,0001	****
CONTROL vs. T1DM +Chitosan	0,0086	**
CONTROL vs. T1DM +Dapagliflozin	0,0449	*
T1DM vs. T1DM +Chitosan	0,0086	**
T1DM vs. T1DM +Dapagliflozin	0,0014	**
T1DM +Chitosan vs. T1DM +Dapaglifloz	0,8982	ns
